# Methylone promotes neurite outgrowth and has long-lasting effects on fear extinction learning

**DOI:** 10.1038/s41386-025-02206-z

**Published:** 2025-08-23

**Authors:** Jennifer Warner-Schmidt, Martin Stogniew, Blake Mandell, Benjamin Kelmendi

**Affiliations:** 1 Transcend Therapeutics, New York, NY USA; 2 Dept. of Psychiatry, Yale School of Medicine, New Haven, CT USA; 3 US Department of Veteran Affairs, National Center for PTSD Clinical Neurosciences Division, West Haven, CT USA

**Keywords:** Cellular neuroscience, Neurotrophic factors

## Abstract

Post-traumatic stress disorder (PTSD) is a prevalent and debilitating disorder, and available treatments are limited. TSND-201 (methylone) is in clinical trials for PTSD, showing potential to have rapid, robust and long-lasting benefit without direct agonist/antagonist activity at 5HT2A. Alterations in structural neuroplasticity are a well-studied mechanism that may underlie both the pathophysiology and treatment of PTSD. Previous work showed that methylone rapidly induced neuroplasticity-related factors in PTSD-relevant brain areas. The current study was undertaken to determine whether methylone affected structural neuroplasticity (e.g., neurite outgrowth) and whether its effects may also be long-lasting. Methylone stimulated neurite outgrowth, specifically increasing the number of branches and the length of the longest neurite per cell in cultured cortical neurons. Methylone’s effect on neurite branching was blocked by inhibitors of monoamine transporters (reboxetine, escitalopram, JHW-007) whereas its effects on the length of the longest neurite per cell were mediated by trkB receptors or mTor signaling. RNA-seq and functional enrichment analyses suggest that methylone has long-lasting effects on factors that mediate neurite outgrowth. Rapid and long-lasting effects of methylone on fear extinction learning and memory were also observed, consistent with the rapid and long-lasting neuroplasticity effects. Reboxetine blocked methylone’s improvement of extinction recall memory, suggesting NET activity is required for methylone’s behavioral effect. Together, this work provides insight into methylone’s mechanism of action and evidence that rapid-acting pharmacotherapies that induce structural neuroplasticity may have potential to treat PTSD.

## Introduction

Post-traumatic stress disorder (PTSD) is a highly prevalent and debilitating disorder affecting an estimated 6-8% of the general population and as many as 25% among groups who have experienced severe psychological trauma [1, 2]. Current treatments for PTSD include psychotherapy and selective serotonin reuptake inhibitors (SSRIs), which are slow-acting and show limited efficacy [3–5].

TSND-201 (methylone) is in clinical trials for the treatment of PTSD (clintrials.gov identifier: NCT05741710). In July 2025, TSND-201 received Breakthrough Therapy Designation from the US Food and Drug Administration, underscoring the unmet medical need for PTSD and highlighting TSND-201’s potential advantage over existing therapies. Methylone has been well-tolerated in multiple human studies [6–10] and demonstrated rapid, robust, and long-lasting beneficial effects in a randomized, placebo-controlled Phase 2 study in individuals with severe PTSD [7, 11]. Consistent with initial clinical findings, animal studies show that methylone has antidepressant-like effects [12, 13] and non-sedating anxiolytic activity [13, 14].

Methylone is a highly selective substrate for the norepinephrine (NE), serotonin (5HT), and dopamine (DA) transporters (NET, SERT, and DAT, respectively) that rapidly increases the release of NE, 5HT and DA [15–21]. Factors that support neuroplasticity are also rapidly increased in the frontal cortex and amygdala [21], two brain areas well-studied for their role in PTSD [2]. Increased neuronal activity, synaptogenesis, and evidence of myelin plasticity in the amygdala may also mediate some of methylone’s behavioral effects [21, 22].

Clinical and preclinical studies illustrate a role for neuroplasticity in the pathophysiology and treatment of PTSD. A large body of evidence collected from human imaging, postmortem, and animal studies highlights the atrophy of neurons, including the retraction of neurites, in the prefrontal cortex in individuals with PTSD and in stress-induced behavioral models [2, 23–26]. The only two FDA-approved treatments for PTSD are SSRI antidepressants (i.e., paroxetine and sertraline). After weeks of daily dosing, SSRIs increase expression of neurotrophic factors, including brain-derived neurotrophic factor (BDNF) [27]. The induction of neurotrophic factors promotes the survival, growth, and function of neurons, modifying synaptic connections and regulating processes like axonal sprouting, dendritic growth, and synaptic strength [28]. Exploratory treatments like MDMA-assisted psychotherapy have also shown clinical benefit for PTSD [29, 30]. Both current and exploratory drugs in clinical development for PTSD (e.g., serotonergic psychedelics, ketamine, and MDMA) have been shown to induce structural remodeling in vivo, in brain areas relevant to PTSD such as the prefrontal cortex [25, 31, 32]. These drugs also increase neurite outgrowth and branching in vitro via BDNF and mTor signal transduction pathways [25, 33]. Such direct effects of methylone on neuroplasticity have not yet been reported.

The current study was undertaken to evaluate whether methylone directly affects neurite outgrowth and if so, to determine which pathways might mediate these effects. Since methylone’s behavioral and clinical effects are long-lasting, we examined whether neuroplasticity-related gene expression changes persist 1-week after dosing and confirmed that methylone-induced effects on fear extinction, an animal model of PTSD, were also long-lasting. We hypothesize that methylone, through rapid release of NE, 5HT, and DA, increases neurite outgrowth and the factors to support neuronal survival in the frontal cortex and amygdala, to support behavioral responses that may be beneficial for PTSD.

## Methods

### Animals

Primary culture studies using E18 embryos derived from pregnant female Sprague Dawley rats (Envigo RMS, Israel) were performed at MD Biosciences (Israel). In-life treatments for RNA-seq studies at WuXi Apptec, Inc. (Cranbury, NJ) used male Sprague-Dawley rats (6–8 weeks old, Hilltop). Fear extinction at Melior Discovery (Exton, PA) used 10-week-old male C57BL/6 mice (Jackson Laboratories). Animals were maintained on a 12 h light/dark cycle with ad libitum access to standard rodent chow and water. Animal use and procedures were in accordance with established protocols approved by the MDBiosciences IACUC committee, MDBiosciences Standard Operating Procedures (SOP), WuXi Apptec, Inc., IACUC, Melior IACUC committee, and Transcend Therapeutics.

### Drugs

Sources and catalog numbers for compounds are as follows: Methylone HCl (Cayman Chemical, #20891), reboxetine (Cayman Chemical, #15038), escitalopram oxalate (Cayman Chemical, #39841) JHW-007 HCl (Tocris, #4351), Ana-12 (Sigma, #SML0209) and rapamycin (Sigma, #R0395).

### Primary cortical cultures

Primary neuronal cultures were derived using standard protocols. Cortex from E18 embryos was removed on ice and dissociated at 37 °C for 20 min in a solution containing 1 ml of Tryple Express solution (Gibco). Following centrifugation (500 RPM, 5 min), supernatant was removed and 1.5 ml neuronal culture medium with serum was added. Single cells were dissociated using glass Pasteur pipettes and plated on prepared coverslips in 24-well plates in brain culture medium supplemented with 5% fetal bovine serum. Cultures were maintained at 37 °C, 5% CO_2_.

### Neurite outgrowth assays

On day-in-vitro 1 (DIV1), cells were treated with methylone HCl (10 µM) or vehicle (0.25% methanol) in neuronal culture medium. For inhibitor studies, Reboxetine (100 nM), Escitalopram (10 nM), JHW-007 (100 nM), Ana-12 (10 µM), Rapamycin (100 nM), or vehicle were applied 10 min before methylone HCl or vehicle on DIV1.

On day-in-vitro 3 (DIV3), cells were fixed with 100% methanol for 20 min, washed 3× with Dulbecco Phosphate Buffered Saline (DPBS, Sartorius), blocked with 5% BSA in PBS and 1% goat serum (Sigma) for 1 h at RT, and washed 3× with DPBS. Cells were incubated overnight with 1:400 Alexa-fluor 594 conjugated beta tubulin III antibody (Abcam) in blocking solution. On day-in-vitro 4 (DIV4), cells were washed with DPBS and coverslips were mounted on slides using fluorescent mounting medium (Immu-Mount, Epredia). Imaging was performed using a ZEISS Observer. Images were analyzed using the NeuroJ plug-in for ImageJ. Total neurite length, the length of the longest neurite per cell, and the number of branches (i.e., the sum of all branches in all neurites of the cell) were calculated for each cell.

### Western blotting

Primary cortical neurons were seeded into poly-L-lysine coated flasks. Following treatment, cells were lysed using 300 microliters of N-per buffer (Thermo Fisher), vortexed periodically while on ice for 20 min, and centrifuged 14,000 RPM for 30 min. Supernatant was collected and total protein concentration was determined using a BCA kit. Samples were loaded onto 4-12% gels, run at 100 V for 10 min followed by 200 V for 30–50 min. Following transfer, membranes were incubated in 5% non-fat dry milk in TBST for 1 h at room temperature, washed with TBST, and incubated with primary antibody at 2–8  °C overnight. Membranes were washed with TBST. Secondary antibody (1:20,000, goat anti-rabbit) in blocking buffer was followed by TBST washes, and membranes were developed using an ECL kit.

### RNA-sequencing

Rats were given methylone (10 mg/kg IP) or vehicle 1w before amygdalae were rapidly dissected and immediately frozen. In-life work was conducted at WuXi Apptec (Cranbury, NJ). RNA extraction, library preparation, sequencing and analysis were conducted at Azenta Life Sciences (South Plainfield, NJ) exactly as described previously [21].

Pathway analysis was performed on selected gene lists based on a statistical and fold-change cutoff (0.32 ≥ log2FC < − 0.32, padj ≤ 0.05) using Metascape gene annotation and analysis resource [34].

### Fear extinction

The behavioral procedure was adapted from [35], with minor modifications. Mice were handled for 2 consecutive days for 4 min/day. Fear conditioning was conducted in automated chambers using infrared beams (Kinder Scientific, Poway, CA). Freezing behavior was recorded in 5 s intervals during sessions on days 1, 3, 4, 8, and 14.

Fear conditioning (D1) consisted of a single CS-US pairing in context A (2 min habituation, 30 s 80 dB tone presentation, 1 mA footshock during the last 2 s of the tone). On day 3, all underwent extinction training, consisting of 4 CS presentations in a novel context (context B, 2 min habituation, 4 × 30 s presentations of the tone, 45 s intertrial interval, no shock). In the first experiment, methylone (10, 20, or 30 mg/kg, IP) or vehicle were administered once on day 3, 30 min before extinction training. On days 4, 8, and 14, extinction recall was probed using 4 CS presentations in context B as performed on day 3, but without drug treatment. In the second experiment, reboxetine, escitalopram, or JHW-007 (all 10 mg/kg, IP) were administered 10 min before vehicle or methylone (10 mg/kg, IP) on day 3, 30 min before extinction training. Extinction recall was probed as described above on day 4.

### Statistical analysis

Two-way ANOVA and post-hoc Tukey’s multiple comparison test were used. For fear conditioning, mixed effects analysis and post-hoc Fisher’s LSD test were used. All analyses were performed using Graphpad Prism software Version 10.0.3. Statistical significance was set at *p* < 0.05.

## Results

### Methylone stimulates neurite outgrowth in vitro

**Fig. 1:**
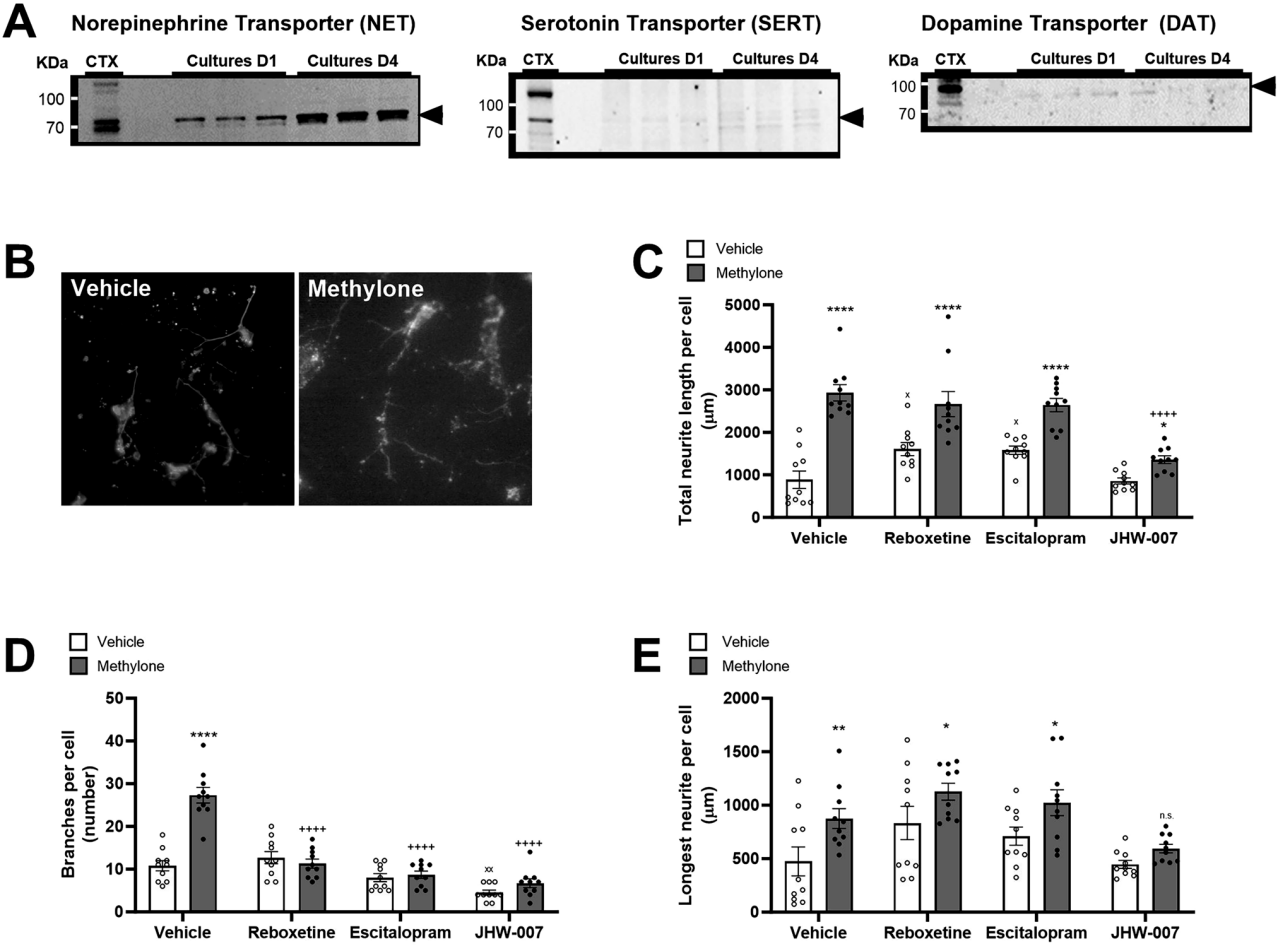
Methylone increases neurite length and branching, at least in part, via activity at monoamine transporters. **A** Western blotting analysis showed that primary cortical neurons express NET, SERT, and DAT on the day of drug application (Cultures D1) and fixation (Cultures D4), although to a lesser extent compared to adult rat cortex (CTX). **B** Representative image of neurons treated with vehicle or methylone (10 μM). **C**–**E** Neurons were pre-treated with vehicle or inhibitors of NET (reboxetine), SERT (escitalopram oxalate), or DAT (JHW-007) before application of either vehicle or methylone. **C** Total neurite length per cell is shown in microns. Statistical analysis of total neurite length showed a significant effect of methylone (F_(1,72)_ = 92.09, p < 0.0001), inhibitors (*F*_(3,72)_ = 15.73, p < 0.0001), and a significant interaction (F_(3,72)_ = 6.987, *p* < 0.001). **D** The number of branches per cell under various treatment conditions is shown. Statistical analysis of the number of branches per cell revealed a significant effect of methylone (F_(1,72)_ = 29.86, p < 0.0001), inhibitors (*F*_(3,72)_ = 50.00, *p* < 0.0001), and a statistically significant interaction (*F*_(3,72)_ = 24.40, p < 0.001). **E** The length of the longest neurite per cell under various treatment conditions is also shown. Statistical analysis showed a significant effect of methylone (F_(1,72)_ = 16.19, p = 0.0001), inhibitors (F_(3,72)_ = 8.077, p = 0.0001) but no significant interaction (F_(3,72)_ = 0.5354, p = 0.66). N = 10 per group; *p < 0.05, **p < 0.01, ****p < 0.0001 vs. respective vehicle group; ^x^p < 0.05, ^xx^p < 0.01 vs. vehicle/vehicle group; ^++++^p < 0.0001 vs. vehicle/methylone group.

Methylone is a highly selective substrate for NET, SERT, and DAT [15, 18]. Before probing methylone’s effects on neurite outgrowth in vitro, we first determined whether each transporter was expressed by the cultured neurons using Western blotting analysis (Fig. 1A). Cultured neurons expressed all three transporters on the day of stimulation (Cultures D1) and the day of fixation (Cultures D4). At both time points, NET was most highly expressed by the cultures, and relatively low levels of SERT and DAT were detected compared with levels detected in adult rat cortex (CTX).

To evaluate direct effects of methylone on neurite outgrowth, we first treated rat E18 primary cortical neurons with methylone (10 micromolar) or vehicle on day 1 and measured neurite lengths and branching on day 3. Methylone significantly increased the total neurite length per cell by more than 3-fold compared to vehicle controls (Fig. 1B, C). Methylone increased the number of branches per cell by 2.5-fold compared to vehicle (Fig. 1D). The length of the longest neurite per cell was also significantly increased by methylone by ~85% compared to vehicle-treated neurons (Fig. 1E).

Next, we evaluated whether the effects of methylone on neurite outgrowth were mediated by NET, SERT, or DAT by pharmacological inhibition of each transporter before the application of methylone or vehicle. Inhibition of NET, SERT, and DAT was achieved using reboxetine (100 nM), escitalopram oxalate (10 nM), and JHW-007 (100 nM), respectively, 10 min before methylone or vehicle application. Overall, methylone’s effect on total neurite length was blunted by JHW-007, but not by reboxetine or escitalopram. In the absence of inhibitors, methylone significantly increased total neurite length by ~300%. Pretreatment with JHW-007 significantly blunted methylone’s effect to ~50% above vehicle controls. Reboxetine and escitalopram, but not JHW-007, significantly increased neurite length by ~80% in the absence of methylone, but did not change the effect of methylone (Fig. 1C). Therefore, the effect of methylone on total neurite length appears to be mediated by its activity at the dopamine transporter.

Changes in neurite outgrowth may be attributed to changes in the number of branches or in the length of longest neurite(s). First, the number of branches per cell was counted in the presence or absence of inhibitors and/or methylone. Pretreatment with reboxetine, escitalopram, or JHW-007 completely abolished the effect of methylone on the number of branches (Fig. 1D). In the absence of methylone, JHW-007 significantly reduced branching compared to vehicle controls.

In contrast to the effects on branching, the effects of methylone on the length of the longest neurite were blunted, but not abolished, by reboxetine, escitalopram, or JHW-007. In the absence of inhibitors, methylone increased the length of the longest neurite by ~80%, but in the presence of reboxetine, escitalopram, or JHW-007, methylone increased the length of the longest neurite by 35%, 43%, or 33% respectively (Fig. 1E). The effect of methylone in the JHW-007 treated group did not reach statistical significance (p = 0.3, n.s.), and there was no effect of any inhibitor in the absence of methylone.

### Effect on the length of longest neurite is blocked by inhibiting neurotrophic factor signaling

**Fig. 2:**
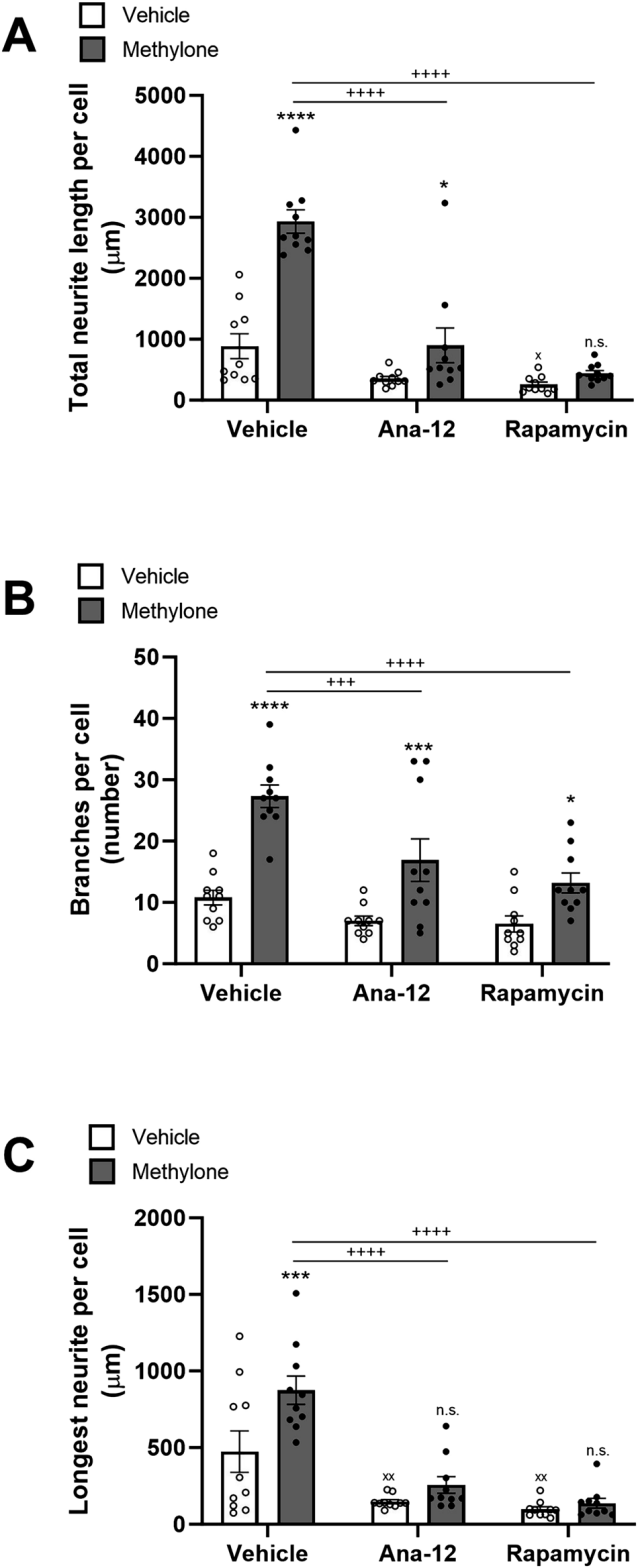
Inhibition of neurotrophic factor signaling abolishes methylone’s effect on the length of longest neurites. Ana-12 and rapamycin were used to inhibitor trkB receptors and mTor signaling, respectively. **A** Total neurite length per cell is shown in microns. There was a significant effect of methylone (F_(1,54)_ = 46.97, p < 0.0001), inhibitors (F_(2,54)_ = 50.42, p < 0.0001), and a significant interaction (F_(2,54)_ = 17.77, p < 0.0001). **B** Number of branches per cell is shown. There was a significant effect of methylone (F_(1,54)_ = 50.60, p < 0.0001), inhibitors (F_(2,54)_ = 12.88, p < 0.0001), and a significant interaction (F_(2,54)_ = 3.460, p < 0.05). **C** The length of the longest neurite per cell is shown. There was a significant effect of methylone (F_(1,54)_ = 9.713, p < 0.01), inhibitors (F_(2,54)_ = 34.74, p < 0.0001), and a significant interaction (F_(2,54)_ = 3.549, p < 0.05). N = 10 per group; *p < 0.05, ***p < 0.001, ****p < 0.0001 vs. respective vehicle group; ^x^p < 0.05, ^xx^p < 0.01 vs. vehicle/vehicle group; ^+++^p < 0.001, ^++++^p < 0.0001 vs. vehicle/methylone group; n.s. = not significant.

Methylone rapidly induces the expression of neurotrophic factors in the cortex, including BDNF [18]. To determine whether neurotrophic factor signaling played a role in methylone’s neurite outgrowth effects, neurons were pretreated with trkB receptor inhibitor (Ana-12, 10 micromolar) or mTor inhibitor (rapamycin, 100 nM) 10 min before methylone or vehicle. Ana-12 and rapamycin significantly reduced methylone’s effects on total neurite length (Fig. 2A). In the absence of methylone, both inhibitors also reduced total neurite length, but rapamycin reached statistical significance (p < 0.05) while Ana-12 did not (p = 0.06).

Ana-12 and rapamycin blunted the effect of methylone on the number of branches per cell, but methylone still significantly increased the number of branches in the presence of the inhibitors (Fig. 2B).

In contrast, Ana-12 and rapamycin completely abolished the effect of methylone on the length of the longest neurite (Fig. 2C). Both inhibitors also significantly reduced the length of the longest neurite per cell in the absence of methylone.

### Biological pathways and functions regulated by methylone are similar in vitro and in vivo

**Fig. 3:**
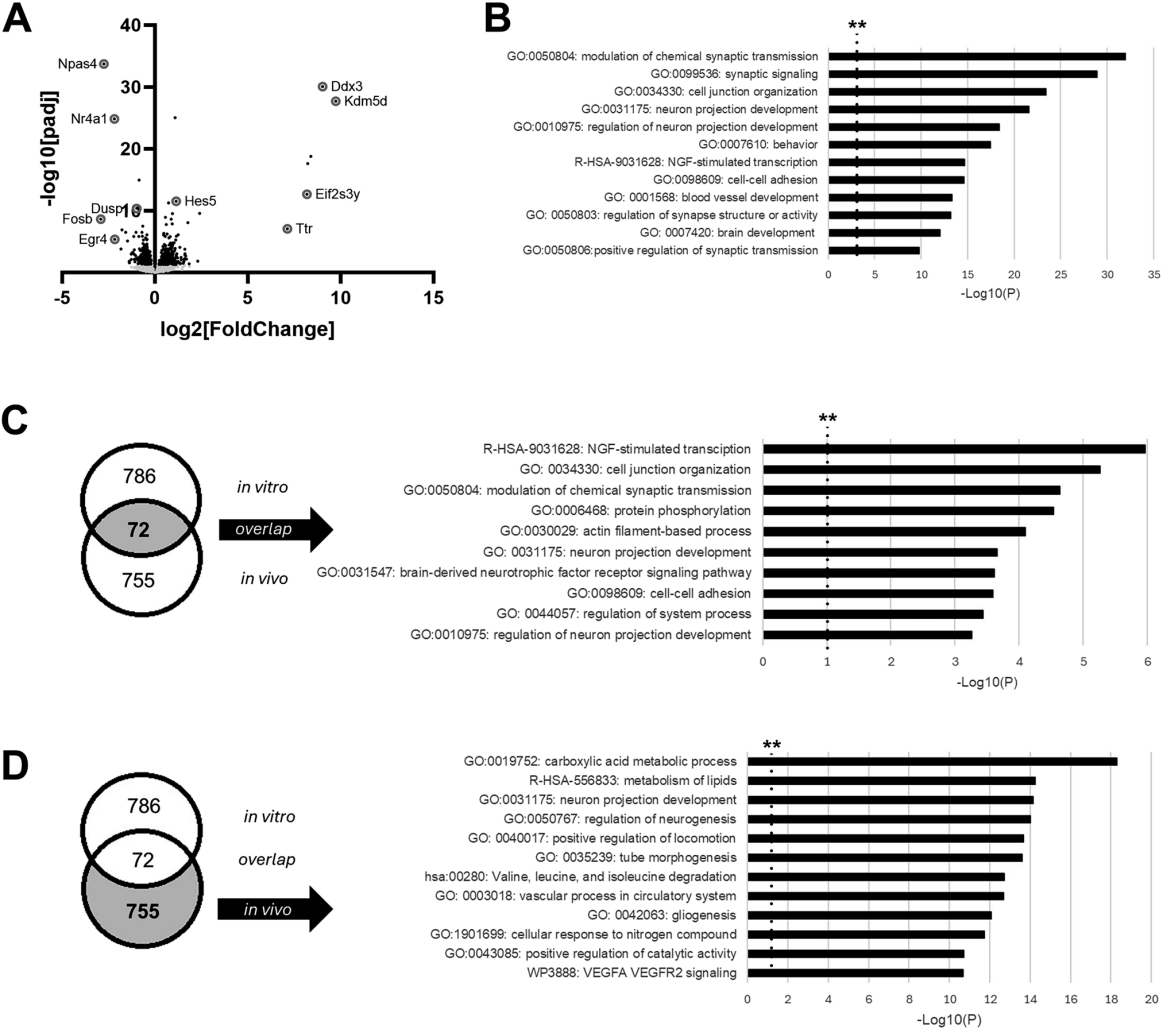
Methylone regulates synaptic transmission and plasticity-related gene expression in vitro. Methylone-induced gene expression changes in vitro are shown. **A** Volcano plot shows significantly regulated genes in cultured neurons after treatment with methylone (black dots) compared with vehicle-treated controls. Light gray dots represent genes that were not significantly changed by treatment. Circled/labeled genes are among the most highly or significantly regulated, and their functions are highlighted in the Discussion. **B** Functional enrichment analysis of genes regulated by methylone compared to vehicle controls show highly significant effects of genes related to synaptic transmission, neuronal development and related biological functions. **C** Comparison with results from previous analysis of rat frontal cortical tissue after methylone administration [18] shows that the two data sets share 72 genes in common. Functional enrichment analysis shows highly significant effects of genes related to NGF- and BDNF-signaling, synaptic transmission, neuronal development, and related biological functions. **D** Functional enrichment analysis of the genes that were induced by methylone in vivo but not in vitro indicated highly significant effects of genes related to carboxylic acid metabolic process, lipid metabolism, gliogenesis, and VEGF signaling. In **B**–**D**, black dashed line with ** marks the −Log10(P) = 1 value where p = 0.01.

We previously observed a rapid increase in neuroplasticity-related gene expression hours after methylone treatment in vivo [21]. To determine whether similar biological pathways and functions were activated when methylone was applied directly to neurons in vitro, here we applied methylone or vehicle to primary neuronal cultures as above and used RNA-seq to assess gene expression changes. Differential expression analysis of the RNAseq data revealed genes significantly regulated by methylone compared to vehicle-treated controls. A total of 858 genes were significantly regulated by methylone (padj ≤ 0.05) and increased or decreased by more than 25% compared to controls (Fig. 3A). Three-hundred ninety-six genes were significantly increased by methylone, and 462 genes were significantly downregulated by methylone. Among the most highly significant and/or down-regulated genes were dual specificity phosphatase 1 (Dusp1), FosB proto-oncogene (Fosb), early growth response protein 4 (Egr4), neuronal PAS domain protein 4 (Npas4), nuclear receptor subfamily 4 group A member 1 (Nr4a1). Among the most highly significant and/or up-regulated genes were DEAD-box helicase 3 (Ddx3), lysine demethylase 5D (Kdm5d), eukaryotic translation initiation factor 2 subunit 3 (Eif2s3y), hes family bHLH transcription factor 5 (Hes5), and transthyretin (Ttr).

Functional enrichment analysis was performed on the 858 genes to determine whether similar biological pathways, functions, and phenotypes were regulated in vitro (i.e., when methylone was applied directly to neurons instead of systemically administered). Results show that the categories identified were related to neuronal projection development, synaptic transmission, and other related functions (Fig. 3B), which appeared categorically similar to genes regulated in vivo (i.e., synaptic signaling, neuron projection development) as reported previously [21].

To determine whether methylone regulated the expression of similar genes in vitro and in vivo, we compared the current in vitro results with results obtained previously following systemic administration of methylone in vivo [21]. DE analysis of RNAseq data from the frontal cortex of rats systemically administered methylone identified ~800 genes differentially expressed compared to vehicle controls (see [21]). Comparison of the in vitro and in vivo gene lists identified 72 genes in common (Fig. 3C). Functional enrichment analysis of those 72 genes showed that NGF-stimulated transcription, BDNF receptor signaling pathway, and functions related to neuronal projections and related processes were among the most significant GO terms (Fig. 3C).

To determine whether there were pathways and functions unique to the systemic administration of methylone in an in-tact brain, we performed functional enrichment analysis on the 755 genes that were regulated by methylone in the in vivo study only (Fig. 3D). Results showed that among the most highly significant GO terms were carboxylic acid metabolic process, metabolism of lipids, neuron projection development, gliogenesis, and VEGF signaling (Fig. 3D).

### Gene expression changes in the amygdala one-week after methylone treatment in vivo

Based on methylone’s long-lasting activity in animals and humans [7, 11–13, 21, 22], we hypothesized that long-lasting changes in gene expression related to neurite outgrowth and/or synaptic transmission may be observed at a longer timepoint after methylone treatment. To test this, rat amygdalae were dissected one week after methylone of vehicle treatment, mRNAs were extracted and analyzed by RNA-seq.

**Fig. 4:**
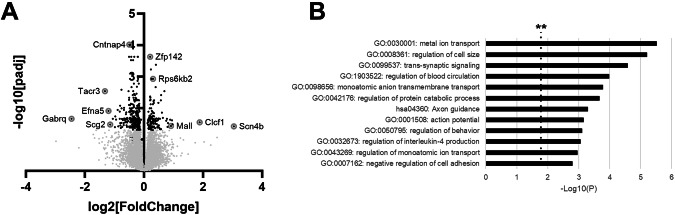
Regulation of neuroplasticity-related gene expression in the amygdala 1-week after methylone treatment. Methylone-induced gene expression changes in the amygdala are shown. **A** Volcano plot shows significantly regulated genes in the amygdala after treatment with methylone (black dots) compared with vehicle-injected controls (N = 6 per group). Light gray dots represent genes that were not significantly changed by treatment. Circled/labeled genes are among the most highly or significantly regulated, and their functions are highlighted in the Discussion. **B** Functional enrichment analysis of genes regulated by methylone compared to vehicle controls show highly significant effects of genes related to ‘axon guidance’ and related biological functions. Black dashed line with ** marks the -log(P) = 1 value where *p* = 0.01.

Differential expression (DE) analysis of the RNAseq data revealed 181 genes significantly regulated by methylone by more than 25% compared to vehicle-treated controls (Fig. 4A). Fifty-two genes were significantly increased by methylone, and 129 genes were significantly downregulated by methylone. Among the most highly significant and/or most downregulated genes (i.e. greatest fold change compared to vehicle) were the theta subunit of the GABA-A receptor (Gabrq), tachykinin receptor 3 (Tacr3, also called NK3R), ephrin A5 (Efna5), secretogranin 2 (Scg2), and contactin-associated protein family member 4 (Cntnap4). Among the most significantly upregulated genes were cardiotrophin-like cytokine factor 1 (Clcf1), zinc finger protein 142 (Znf142), T Cell Differentiation Protein Like (Mall), the beta-subunit 4 of the voltage-gated sodium channel (Scn4b), and ribosomal protein S6 kinas b2 (Rps6kb2).

Functional enrichment analysis was performed on the 181 genes to classify the list of methylone-regulated genes and associate them with biological pathways, functions, or phenotypes (Fig. 4B). Most of the categories identified suggest functions related to neuronal activity and axon guidance. Among the most significant GO terms were metal ion transport, regulation of cell size, trans-synaptic signaling, axon guidance and action potential.

### Methylone had a long-lasting effect on fear extinction learning and memory

**Fig. 5:**
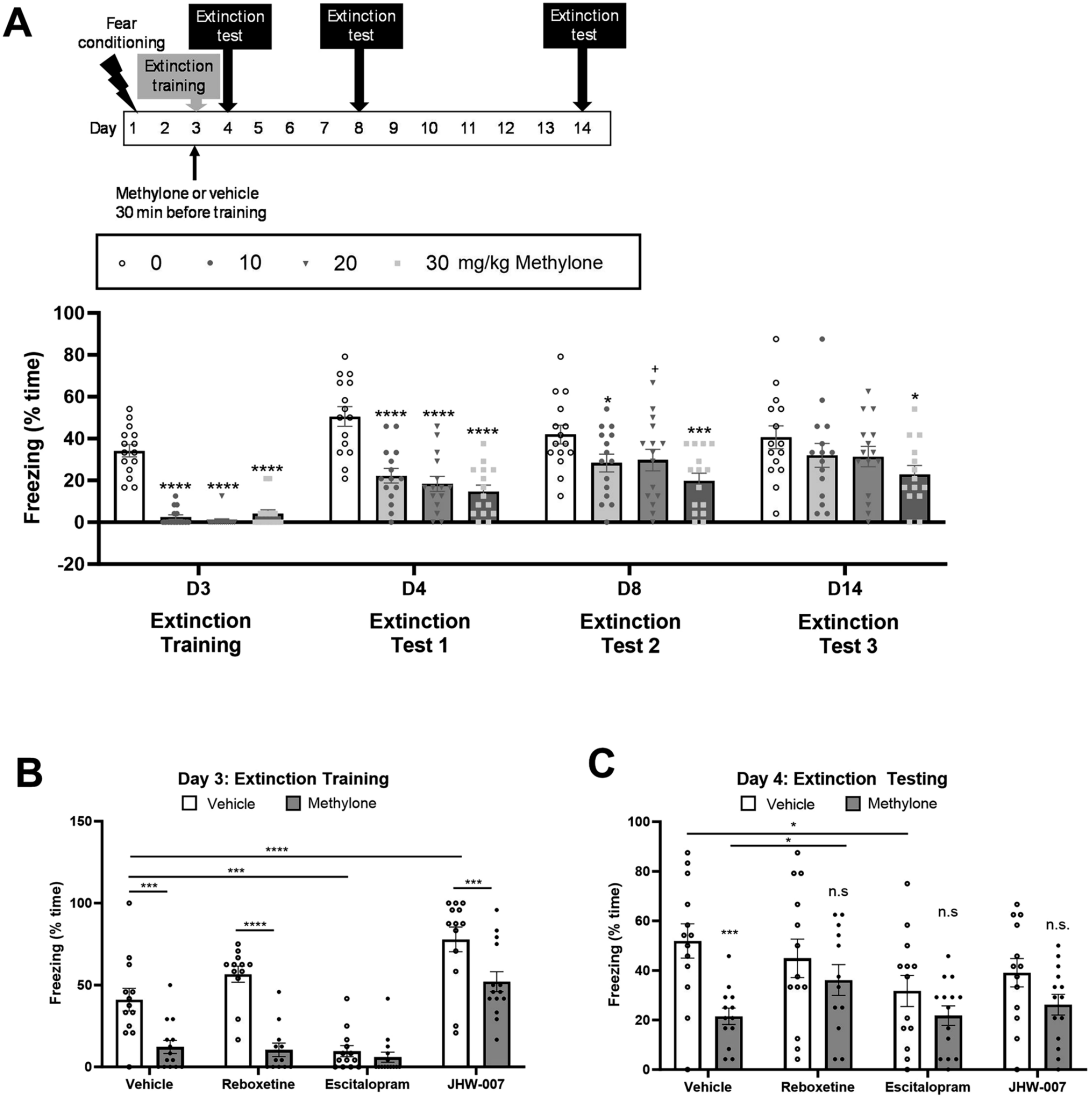
Methylone’s effect on fear extinction learning and memory is long-lasting and blocked by NET inhibition. **A** Schematic of experimental design (top). Time spent freezing during the extinction training session on day 3 (D3) and during extinction tests performed on days 4, 8, and 14 (D4, D8, D14) (bottom). Statistical analysis showed a significant effect of methylone (F_(3,56)_ = 16.78, p < 0.0001), time (F_(2.528,140.7)_ = 37.73, p < 0.0001), and a significant interaction (F_(9,167)_ = 3.511, p < 0.0001). N = 14–15 per group; *p < 0.05, ***p < 0.001 ****p < 0.0001, +p = 0.08 vs. vehicle control group. **B**, **C** To evaluate the effect of NET, SERT, and DAT activity on methylone’s effects on fear extinction learning and memory, animals were pretreated with reboxetine, escitalopram oxalate, or JHW-007 (10 mg/kg for each) 10 min before vehicle or methylone (10 mg/kg) treatment. Extinction training took place 30 min post-dose with methylone or vehicle. **B** Time spent freezing on day 3 is shown. Statistical analysis showed a significant effect of methylone (F_(1,97)_ = 49.38, p < 0.0001), inhibitors (F_(3,97)_ = 42.30, p < 0.0001), and interaction (F_(3,97)_ = 5.404, p < 0.01). N = 12-14 per group; ***p < 0.001; ****p < 0.0001. **C** Time spent freezing during extinction testing on day 4 ( ~ 24 h post-dose) is shown. Statistical analysis showed a significant effect of methylone (F_(1,97)_ = 15.03, p < 0.001), trend for inhibitors (F_(3,97)_ = 2.152, p < 0.1), and no significant interaction (F_(3,97)_ = 15.03, p = 0.2). N = 12-14 per group; ***p < 0.001 vs. vehicle/vehicle group; n.s. not significant vs. respective vehicle control; *p < 0.05 as noted.

Next, we hypothesized that the observed rapid and long-lasting changes in neuroplasticity would correlate with rapid and long-lasting changes in an animal model of PTSD. Consistent with previous studies [22, 35], mice underwent fear conditioning, and the effect of methylone on extinction learning and recall memory was evaluated. Methylone or vehicle were administered 30 min prior to extinction training (D3) and extinction testing was done on days 4, 8, and 14 (Fig. 5A). All doses of methylone tested (10, 20, 30 mg/kg) significantly reduced freezing to the CS during extinction training on day 3 by 88-98% compared to vehicle controls (Fig. 5B, Figure S1). Extinction recall testing on day 4 demonstrated that all doses tested significantly reduced freezing to the CS by 60-70%. Effects of the lower doses (10, 20 mg/kg) persisted through day 8 (~30%), but the highest dose tested (30 mg/kg) significantly reduced freezing on day 14 by 44% compared to vehicle controls (Fig. 5B).

To evaluate whether effects of methylone on fear extinction learning and memory were mediated by its activity at monoamine transporters, rats underwent fear conditioning as described above, but were injected with inhibitors of NET, SERT, or DAT (reboxetine, escitalopram, or JHW-007, respectively), 10 min prior to treatment with methylone or vehicle. Inhibitors did not alter methylone’s activity on extinction training on day 3 (Figs. 5C and S2). Escitalopram alone significantly reduced freezing, comparable to methylone’s effect, though this may be due to transient locomotor stimulation (Fig. S2). On day 4, reboxetine significantly blocked the effect of methylone on fear extinction recall (Fig. 5D) without affecting locomotion (Figure S2). Escitalopram or JHW-007 pretreatment did not alter methylone’s effect, but freezing levels in the escitalopram + methylone and JHW-007 + methylone groups did not differ significantly from their respective vehicle controls (Fig. 5D).

## Discussion

Methylone, which has shown rapid, robust, and long-lasting beneficial effects in humans with PTSD [7, 11] and corresponding antidepressant-like and anxiolytic effects in animals [12, 13, 22], rapidly increases the expression of neuroplasticity-related factors in the frontal cortex. Here we demonstrate that methylone directly increases neurite outgrowth, branching, and the length of the longest neurite per cell when applied to primary cortical cultures in vitro*¸* and that these effects can be inhibited by blocking monoamine transporters or neurotrophic factor (i.e., BDNF, mTor) signaling.

Three metrics were measured: total neurite length, number of branches per cell, and length of the longest neurite per cell. Blocking NET, SERT, or DAT individually prevented methylone from increasing branch number, indicating that its activity at all three transporters was required for this effect. Total neurite length, which includes all branches, and the length of the longest neurite per cell were increased by methylone, but only blocked by the DAT inhibitor JHW-007—not by SERT or NET inhibitors—suggesting methylone’s activity at DAT may drive neurite elongation. Methylone’s effect on the length of the longest neurite per cell also required BDNF signaling through trkB and/or mTOR. This may still be due to methylone-induced release of NE, 5HT, and DA, which are known to increase BDNF [21, 36–38], but could also be due to direct effects at trkB similar to fluoxetine, ketamine, and psychedelics [39, 40], which would be interesting to investigate in the future.

Alterations in structural neuroplasticity, and more specifically the atrophy of cortical neurons in the prefrontal cortex may be a common feature of many stress-related disorders, including PTSD [2, 23, 41]. Available treatments for PTSD, specifically SSRIs, require weeks of daily dosing to increase neurotrophic factors (e.g., BDNF), which are believed to mediate neurogenic and therapeutic effect of these drugs [24]. Rapid-acting drugs such as ketamine or others in development (e.g., serotonergic psychedelics and entactogens) increase expression of neurotrophic factors and support neuroplasticity on a rapid time scale, consistent with the time-course of their therapeutic effects [42]. Effects of rapid-acting antidepressants on structural plasticity have mainly been attributed to increased dendritic complexity and spines [31]. Consistent with this, psychedelics have been shown to increase branching and dendritic complexity, but do not affect the length of the longest neurite [25]. Ketamine significantly increased total neurite length, which appeared to be driven mainly by the increased length of the longest dendrite, although the effect did not reach statistical significance [25]. Our results show that methylone increases both branching and the length of the longest neurite in vitro. In future studies, it will be important to validate that effects on dendritic and axonal arborization are also observed in vivo.

Neuronal projections between the medial PFC and the amygdala are posited to play an important role in PTSD pathophysiology and treatment [43, 44]. Gene expression changes consistent with neuroplasticity in the frontal cortex are observed within hours of methylone treatment [21]. Here we show that one week later, gene expression changes related to neurite outgrowth and synaptic transmission are observed in the amygdala. These include known regulators of axon guidance processes including Tacr (also called NK3R) [45], Efna5 [46], and Rps6kb [47]. Cntnap4 is a member of a family of proteins that play important role in myelinated axons [48], which is interesting given earlier evidence of myelin plasticity in the amygdala after methylone treatment [21], the potential role of activity dependent myelin plasticity in shaping neuronal connections [49, 50], and myelin changes observed in PTSD [51]. Long-lasting changes in neuronal connectivity and synaptic transmission may explain how methylone, with a short half-life, has long-lasting effects on animal behavior [12, 13, 22] as well as durable efficacy in a Phase 2 clinical study of individuals with PTSD [7, 11].

RNAseq analysis of cultured neurons treated with methylone was used to investigate functions and pathways that may be regulated downstream of methylone’s primary pharmacology. We found that the most highly and/or significantly regulated genes appeared to code for proteins involved with neuronal and synaptic plasticity that underlie learning and memory function. For example, Dusp1 plays a role in the regulation of synaptic plasticity, neuronal morphology, and neuroprotection [52] and is a transcriptional target of BDNF and glucocorticoids [53, 54]. Fosb is a critical transcription factor that plays a role in cell growth and proliferation and is well-studied role in antidepressant-induced plasticity and stress responses [55] and has been described as a ‘molecular switch for long-term adaptation in the brain’ [56]. Egr4 and other family members that were also regulated (e.g., Egr1, Egr2, Egr3) are transcriptional regulators that play a critical role in learning and memory processes [57]. Npas4 is a transcription factor that regulates the formation and maintenance of inhibitory synapses in response to excitatory synaptic activity affecting the circuitry underlying long-term memory [58]. Nr4a1 plays a role in the regulation of spine density in pyramidal neurons [59]. All of these genes were downregulated in response to methylone treatment, but some care should be taken in the interpretation of the direction of regulation of different factors in cultured neurons 24 h after methylone application, since robust downregulation of gene expression may be a compensatory effect due to increased protein production [60]. However, we may speculate that methylone-induced down-regulation of immediate early genes (IEGs) may reflect a decrease in neuronal activity [61]. Decreased activity may be consistent with methylone’s observed efficacy in PTSD since PTSD has been associated with overactivity of cortico-amygdalar circuitry [2] and hyperactivity of the salience network [62]. Normalizing cortico-amygdalar activity has been suggested to improve emotional engagement, processing of trauma and calm hyperarousal [63]. Future work will be required to address this hypothesis in humans and animal models.

The most highly significantly up-regulated genes included Ddx3, essential for neurite outgrowth [64]; Kdm5d, a less-studied member of a family of histone demethylases that play a role in synaptic structure and function [60]; Eif2s3y, which can regulate synaptic transmission and social behavior [65]; Hes5, an essential component of the Notch pathway, which regulates cellular differentiation [66]; and Ttr, which has been shown to be potentially neuroprotective [67]. Taken together, all of the genes most highly and/or significantly regulated by methylone appear to play some role in neuronal and/or synaptic plasticity functions.

Additional genes regulated only in vivo, and not in vitro, included processes such as carboxylic acid metabolic process, gliogenesis and VEGFA-VEGFR2 receptor signaling. These functions play a crucial role in energy production and neurotrophic support. This is also of interest due to the role of glia [68] and endothelial cells/VEGF [69, 70] in the actions of classic and rapid-acting antidepressants and highlight potential effects of methylone on non-neuronal cell-types in the intact brain.

Finally, deficits in fear extinction have been observed in patients with PTSD and some even conceptualize PTSD as a defect in the extinction of conditioned fear [71], although the evidence for impaired fear extinction in PTSD is mixed [72–75]. In the current study, we confirm that methylone facilitates fear extinction learning and memory [22] and that its effect is both rapid and long-lasting. We cannot exclude the possibility that locomotor stimulating effects may influence results in the fear extinction model. However, methylone has a short half-life in humans (~6 h) and other animals (~1.5-2 h) [8, 13], but its clinical and behavioral effects persist long after the drug leaves the body [7–13, 22]. Neuroplasticity remains a mechanism by which an acute treatment could lead to lasting effects.

Pretreatment with the selective NET inhibitor reboxetine, but not SERT or DAT inhibitors (escitalopram or JHW-007, respectively), blocked the effect of methylone on fear extinction recall memory (24 h post-dose). It is notable that noradrenergic signaling is known to facilitate fear extinction in the context of PTSD [76–79]. However, MDMA effects on facilitation of fear extinction have been attributed to SERT [80]. This suggests that despite their structural similarity, methylone and MDMA may act through different primary mechanisms, consistent with differences in their pharmacology and subjective effects [8–10, 21].

Current and exploratory treatments for PTSD, including SSRIs, MDMA, and serotonergic psychedelics like psilocybin facilitate fear extinction [35, 81, 82] and affect neuroplasticity [25, 31, 32, 83, 84]. Data suggest that 5HT2A receptors mediate these effects. However, methylone does not have direct agonist/antagonist activity at 5HT2A receptors [19, 21]. Therefore, these drugs may affect the underlying fear neurocircuitry via different primary targets.

In conclusion, methylone’s effects on neuroplasticity and the factors that support it help to explain how a drug with such a short half-life can provide durable effects in humans and animal models of PTSD. These results reinforce the potential for methylone as a treatment for PTSD and, more generally, offer evidence towards elucidating mechanistic effects that are relevant to the benefit of rapid-acting pharmacotherapies.

## Supplementary information

Figure 5 Supplemental Material

## Data Availability

The datasets generated during the current study will be deposited in NCBI’s Gene Expression Omnibus (GEO Series accession number pending) upon acceptance and before publication of this manuscript. The previously published data discussed in have been deposited in NCBI’s Gene Expression Omnibus (Warner-Schmidt et al., 2024) and are accessible through GEO Series accession number GSE253280 (https://www.ncbi.nlm.nih.gov/geo/query/acc.cgi?acc=GSE253280).
