# Supplementary material for: Methylone promotes neurite outgrowth and has long-lasting effects on fear extinction learning
Source: Neuropsychopharmacology. 2025 Aug 23;51(3):631–40. doi: 10.1038/s41386-025-02206-z (PMC12823647; doi:10.1038/s41386-025-02206-z)

Figure S1. Freezing to conditioned stimulus presentations and baseline activity for fear extinction experiment 1. (A) Freezing during each of the four conditioned stimulus presentations on day 3 are shown. (B) Freezing during each of the four conditioned stimulus presentations on day 4 are shown. (C) Baseline activity (counts) during the 2min acclimation period before CS presentations on day 3 is shown. ($F_{(3,56)}$=17.65, $p<0.0001$) (D) Baseline activity (counts) on day 4 is shown. ($F_{(3,56)}$=5.917, $p<0.01$). N=12-14 per group. *$p<0.05$; **$p<0.01$, ****$p<0.0001$, n.s. = not significant as indicated.

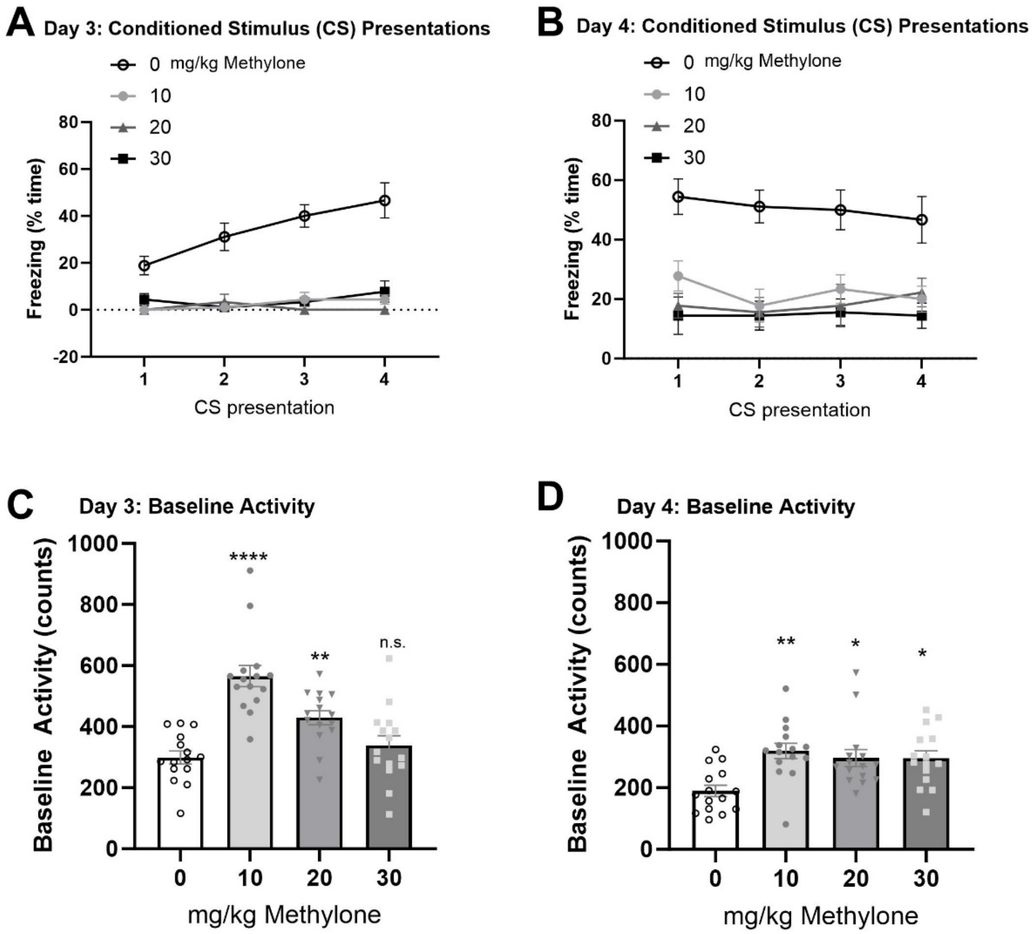

Figure S2. Freezing to conditioned stimulus presentations and baseline activity for fear extinction experiment 2. (A) Freezing during each of the four conditioned stimulus presentations on day 3 are shown. (B) Freezing during each of the four conditioned stimulus presentations on day 4 are shown. (C) Baseline activity (counts) during the 2min acclimation period before CS presentations on day 3 is shown. Statistical analysis showed a significant effect of methylone ($F_{(1,98)}=7.491$, $p<0.01$), inhibitors ($F_{(3,98)}=80.32$, $p<0.0001$), and a trend for interaction ($F_{(3,98)}=2.497$, $p<0.1$). (D) Baseline activity (counts) on day 4 is shown. Statistical analysis showed no significant effect of methylone ($F_{(1,98)}=0.1618$, $p=0.68$), and a trend for an interaction ($F_{(3,98)}=1.878$, $p=0.1$). N=12-14 per group. *p<0.05; **p<0.01, ***p<0.001, ****p<0.0001 as indicated.

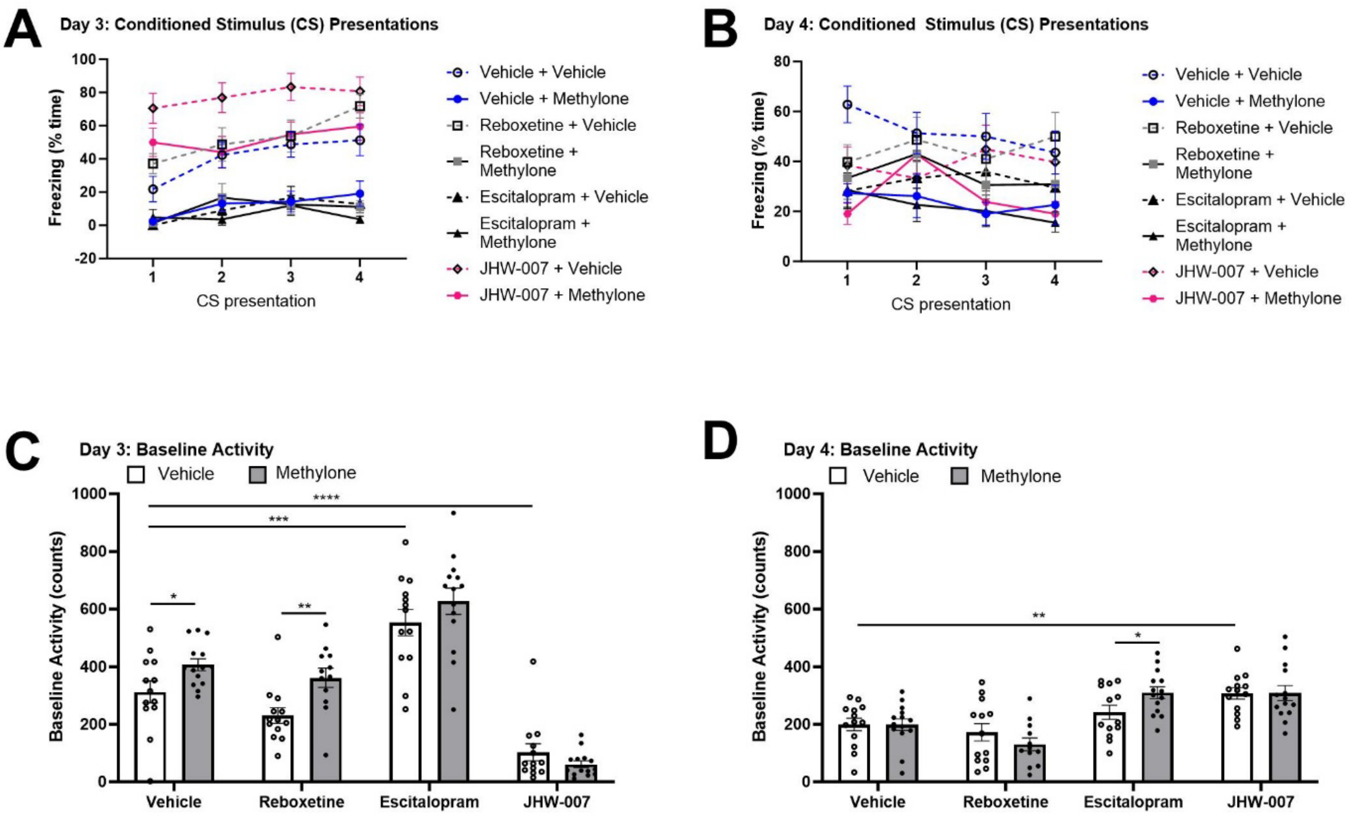

Supplement: Supplementary file 1 — Figure 5 Supplemental Material [file 41386_2025_2206_MOESM1_ESM.pdf]
